# Water Use and Treatment in Container-Grown Specialty Crop Production: A Review

**DOI:** 10.1007/s11270-017-3272-1

**Published:** 2017-03-21

**Authors:** John C. Majsztrik, R. Thomas Fernandez, Paul R. Fisher, Daniel R. Hitchcock, John Lea-Cox, James S. Owen, Lorence R. Oki, Sarah A. White

**Affiliations:** 1 Department of Plant and Environmental Sciences, Clemson University, E-143 Poole Agricultural Center, Clemson, SC 29634-0310 USA; 2 Department of Horticulture, Plant and Soil Science Building, Michigan State University, East Lansing, MI 48824 USA; 3 Environmental Horticulture Department, University of Florida, 2549 Fifield Hall, PO Box 110670, Gainesville, FL 32611-0670 USA; 4 Department of Agricultural Sciences, Baruch Institute of Coastal Ecology and Forest Science, Clemson University, Georgetown, SC 29440 USA; 5 Department of Plant Sciences and Landscape Architecture, University of Maryland, 2120 Plant Sciences Bldg., College Park, MD 20742-4452 USA; 6 Department of Horticulture, Hampton Roads Agricultural Research and Extension Center, Virginia Polytechnic Institute and State University (Virginia Tech), 1444 Diamond Springs Road, Virginia Beach, VA 23455 USA; 7 Department of Plant Sciences, University of California, Davis, One Shields Avenue, Davis, CA 95616-8780 USA

**Keywords:** Water quality, Water conservation, Water treatment, Remediation, Greenhouse, Irrigation, Nitrogen, Nursery, Phosphorus, Plant pathogens, Sediment

## Abstract

While governments and individuals strive to maintain the availability of high-quality water resources, many factors can “change the landscape” of water availability and quality, including drought, climate change, saltwater intrusion, aquifer depletion, population increases, and policy changes. Specialty crop producers, including nursery and greenhouse container operations, rely heavily on available high-quality water from surface and groundwater sources for crop production. Ideally, these growers should focus on increasing water application efficiency through proper construction and maintenance of irrigation systems, and timing of irrigation to minimize water and sediment runoff, which serve as the transport mechanism for agrichemical inputs and pathogens. Rainfall and irrigation runoff from specialty crop operations can contribute to impairment of groundwater and surface water resources both on-farm and into the surrounding environment. This review focuses on multiple facets of water use, reuse, and runoff in nursery and greenhouse production including current and future regulations, typical water contaminants in production runoff and available remediation technologies, and minimizing water loss and runoff (both on-site and off-site). Water filtration and treatment for the removal of sediment, pathogens, and agrichemicals are discussed, highlighting not only existing understanding but also knowledge gaps. Container-grown crop producers can either adopt research-based best management practices proactively to minimize the economic and environmental risk of limited access to high-quality water, be required to change by external factors such as regulations and fines, or adapt production practices over time as a result of changing climate conditions.

## Introduction

The focus of this review is on water use and water recycling in container-grown production of greenhouse and nursery specialty crops. The majority of information and insights in this review also have applicability to containerized edible crops grown in open air or under protected culture. In container-grown crop production, water application frequency varies from multiple times per day to once every few days depending on the production system, crop producer, growing season, and environmental conditions, such as rainfall. Use of containers has grown in popularity with nursery growers over the past 50 years because crops can be produced more rapidly and economically (Majsztrik et al. 2011; U. S. Department of Agriculture 2007) and the root zone (substrate, fertilizer, and water) is easier to modify when compared with field production (Ruter 1993). Ruter (1998) showed that total biomass increased by 27% by growing *Betula nigra* under pot-in-pot conditions compared with aboveground container production, which was likely due to more favorable root zone conditions. Container-grown plants also weigh less and therefore are easier to move and ship, allowing more flexibility at an operation and improving shipping efficiency.

Containerization allows growers to sell plants throughout the year regardless of soil conditions or plant growth stage, which increases productivity per unit area. Field operations typically apply lower rates of fertilizer and water on a per meter or per hectare basis compared with container production because soil matrices are typically more chemically and water buffered (Bailey et al. 1999). Field production also has wider plant spacing (1480 to 12,360 plants per hectare) compared to both container production in nurseries (17,300 to 247,000 plants per hectare) and greenhouses (99,000 to 865,000 plants per hectare) (Majsztrik 2011). As inventories are sold, containerized plants can be consolidated to make room for additional plants, while field operations cannot be consolidated. This greater density (number of plants per unit area) of ornamental container-grown crop production results in both higher revenue and increased material and input costs compared with field production.

Producers of containerized plants face several challenges related to water use and runoff. Irrigation must be applied more frequently in containerized production systems compared to field soils, because plant available water is lower within containers filled with soilless substrates, which have high porosity and restricted root volumes (Allaire-Leung et al. 1999; Argo 1998; Beeson 2007; Owen and Altland 2008). Any water, or agrichemicals (i.e., fertilizers, pesticides, and plant growth regulators) applied in excess of the capacity of the container, are unable to be utilized by the plant, or fall outside of the container will likely leach and run off and may eventually impact surface water and groundwater (Cabrera 1997). Concerns persist that as runoff (i.e., non-point source) leaves an operation, sediment and agrichemical contaminants will also be exported (Berghage et al. 1999; Braden and Uchtmann 1985; Vymazal and Březinová 2015). Some growers capture and reuse all or a portion of production runoff, whereas other growers allow runoff to drain from their operations to the surrounding ecosystem.

Grower hesitation to capture and reuse runoff can usually be attributed to a reluctance to change practices because of concerns about the opportunity cost of lost production area, installation costs of containment and treatment systems, management costs for treatment technology, reintroduction of disease-causing organisms or plant growth regulators, phytotoxicity of reintroduced pesticides, or land characteristic restrictions (high water table, steep slopes, etc.) (White et al. 2013). In this review, we will discuss these challenges, as well as potential solutions to these issues and limitations.

### Operation Types and Irrigation Characteristics

Greenhouses are typically characterized as covered or enclosed systems with the capacity to control environmental factors that impact plant growth, including temperature, humidity, irrigation, and light. Operation sizes typically range from a few hundred square meters to 5 ha but can exceed 10 ha. Greenhouse operations tend to be highly intensive production systems on a per unit area basis, but due to smaller container sizes are typically smaller than container-nursery operations. They typically use precise irrigation applications and can have a high degree of environmental monitoring and control. Thus, greenhouse operations typically require less water per unit area than open-air container or pot-in-pot nurseries (Bailey et al. 1999). This higher degree of control capability can lead to higher distribution uniformity and water use efficiencies. However, efficiencies also depend on irrigation application method (e.g., boom, drip emitters, micro-emitters, or spray stakes), application decisions (irrigation scheduling), and system design and maintenance. The typical higher efficiency irrigation used in greenhouse operations requires higher-quality water (typically via filtration) and regular maintenance to avoid emitter clogging and subsequent plant loss or damage.

Nursery container operations (open-air) place containers at or below ground level (i.e., pot-in-pot). Plants are grown on various combinations of bare ground, gravel, landscape fabric, or other surfaces that are often graded to reduce standing water directly below containers. Nursery container operation sizes can vary from less than a hectare to thousands of hectares. Irrigation is typically applied overhead using impact sprinkler heads or similar-type heads. Larger containers (typically 19 L or larger) are often irrigated using micro-irrigation via drip emitters or spray stakes. Although micro-irrigation is more labor intensive to maintain, the necessity of wider plant spacing due to canopy size makes overhead irrigation inefficient due to wind drift and decreased interception efficiency (more droplets hit the ground instead of a container as spacing increases). Micro-irrigation allows for precise delivery of water to the container-plant system and provides the potential to implement fertigation (irrigation and water-soluble fertilizer applied in unison) if controlled release fertilizers (CRFs) are not used or are depleted before the end of the growing season.

### Water Use in Agriculture

Freshwater is a finite resource. Yet, demand for water has increased due to population growth and increasing water use by agricultural systems needed to support larger populations (Rijsberman 2006). Although most nursery and greenhouse crops do not feed people directly, these plants can enhance human well-being and expand our connection to the natural environment (Kuzevanov and Sizykh 2006; Park et al. 2004). Globally, agriculture is estimated to use 69% of freshwater supplies, while industry and energy use is 23% and household consumption is 8% (O’Neill and Dobrowolski 2011; Santos Pereira et al. 2009). Concerns regarding water scarcity, particularly in arid or semi-arid regions such as the western USA and Australia, intensify during times of drought, but long-term water use continues to be a major problem.

The majority of the specialty crops, grains, fruits, vegetables, and nuts consumed within USA and exported around the world are produced in the western USA (U.S. Department of Agriculture 2014). During times of drought, allocation and conservation of a limited water supply among agriculture, industry, and household use receive increased attention. During 2015–2016, much of California was in either extreme or exceptional drought, the two highest categories, impacting over 36 million people in the state (U.S. Department of Agriculture 2015). Growers were forced to fallow land and remove established agricultural specialty crops because of limited water availability. Changing weather patterns can significantly impact both crop yield in non-irrigated land and the volume of water required to supplement rainfall in irrigated lands (Schlenker et al. 2006). Agricultural systems, in general, will likely need to produce more plants with less water, use lower-quality water, or both (Fulcher et al. 2016).

Crop water use efficiency, defined as the water volume required to produce a given dry mass of yield, and water use reduction can be accomplished in part by breeding for drought tolerance (Bolaños and Edmeades 1996; Cattivelli et al. 2008), but growers must also conserve water through irrigation and other management practices (Beeson et al. 2004; Beeson and Haydu 1995; Biernbaum 1992; Fereres et al. 2003; Fulcher et al. 2016; Lea-Cox 2012; Lea-Cox et al. 2013; Mathers et al. 2005; Pershey et al. 2015; Warsaw et al. 2009a). Increased crop water use efficiency can be achieved via precise water quantity delivery to the container (e.g., sensor or climate modeling-based approaches) based on crop-based demand to limit leaching from over-irrigation. Additionally, irrigation type (Beeson and Knox 1991; Grant et al. 2009; Klock-Moore and Broschat 2001; Lamack and Niemiera 1993), timing (Beeson 1992; Devitt et al. 1994; Grant et al. 2009; Scheiber and Beeson 2007; Tyler et al. 1996a, b; Warren and Bilderback 2004), and use of new technology (Beeson 2005; Ingram and Fernandez 2012; Lichtenberg et al. 2013; Sharp 2007; Shrestha and Gopalakrishnan 1993; van Iersel et al. 2013; Warsaw et al. 2009b) have been reported to increase irrigation efficiency. Regardless of method, improved water application and scheduling precision reduces the presence of agrichemicals and other contaminants in production runoff (Briggs et al. 1998; Million et al. 2007a, b; Pershey et al. 2015; Warsaw et al. 2009a).

### Contaminants in Irrigation Runoff Water

Transport of contaminants from irrigation runoff into the neighboring ecosystem is a concern for all agricultural production, but particularly in specialty crop production (Braman et al. 2015; McCobb et al. 2003; Meador et al. 2012; Vymazal and Březinová 2015; Weston and Lydy 2010). Contaminants of concern in specialty crop operations (e.g., sediment, fertilizer, pesticides, and phytopathogens) can either be removed, recycled on-site, volatilized, or transported off-site, depending upon production practices at the operation and prevailing environmental conditions. Contaminant presence, along with increased economic and regulatory pressure to develop alternative irrigation water sources, results in a challenge for many growers. Recycling runoff water for irrigation is an ideal solution from a water quantity standpoint, in that the water is already available on-site, reducing volume of water needed from other sources. This recycled water also contains contaminants that could be detrimental to the environment; recycling water would help to limit agrichemical escape into the environment (Bailey and White 1964; Karthikeyan et al. 2004; Popov et al. 2006; Zabik et al. 1976). Growers are typically concerned about negative impacts of bioactive concentrations of pesticides or phytopathogens which may diminish crop health if they are present in recycled runoff water. Perception of risk associated with these contaminants represents a significant barrier to grower adoption and use of this readily available water source (White et al. 2013).

Fertilizers deliver plant essential mineral nutrients to ensure optimal growth, but application of fertilizers in excess of plant requirements can result in nutrient leaching; of particular environmental concern are nitrogen (N) and phosphorus (P). Fertilizer runoff from agriculture, including specialty crop production, is a major problem in a number of impaired waterways and can lead to environmental problems such as algal blooms (Majsztrik and Lea-Cox 2013; Mangiafico et al. 2009; White 2013a). The ability to recycle mineral nutrients is perceived as a benefit for some growers, and these recycled fertilizer salts are sometimes accounted for in their nutrition programs, particularly in greenhouse production (White et al. 2013).

Agrichemical residues in water can be detrimental if not mitigated, as both surface water and groundwater can become contaminated (Briggs et al. 2002). The fate and transport of agrichemicals depends on a number of factors, including location applied, soil characteristics, slope, and timing of rain/irrigation events (Lagaly 2001; McGechan and Lewis 2002; Wauchope 1978). Chemicals vary in their modes of action and half-lives in the environment (Calderbank 1989; van der Werf 1996); thus, managing agrichemical contaminants in recycled runoff can be challenging. However, prevention of contamination and remediation of contaminants to minimize reapplication injury to the crop and environmental/biotic damage is feasible using best management practices (BMPs).

Phytopathogen contamination can create economic and ecosystem stressors, causing disease within both the operation and the surrounding ecosystem via runoff (MacDonald et al. 1994). Economic analysis of production losses attributed to phytopathogens in container-grown specialty crops is not widely available, making it difficult to calculate the impact on grower profits and the surrounding environment. Specialty crop production losses to pathogen infection have been estimated to range from 5 to 30% for some crop taxa, but losses are likely to be crop specific and fluctuate annually based on environmental and production conditions (Chappell et al. 2012; Loyd et al. 2014; Williams-Woodward et al. 2009). Ecosystems may be negatively impacted by the discharge of pathogens from crop production facilities via plant transport from nurseries and eventual pathogen escape into the environment as illustrated by the pathogen causing sudden oak death, *Phytophthora ramorum* (Gruenwald et al. 2008; Sansford et al. 2008). While fungicide applications can suppress pathogen growth, in general they are not curative. As a result, many growers prefer to minimize potential for crop infection by either sanitizing water before it is used (e.g., chlorination) or not reusing runoff. Management of pump intake depth and location within a reservoir were identified by Ghimire et al. (2011) as key mechanisms for limiting introduction of pathogen propagules via irrigation water. Additional insights into propagule movement, survival, persistence, and/or pathogenicity in production runoff and their economic and environmental impacts are potential areas of future study.

### Impaired Waters of the USA

In 1972, the USA passed the Clean Water Act, which created an impaired waters list [also known as the 303(d) list], which identifies bodies of water that do not meet water quality standards, including chemical contaminants, dissolved oxygen, excess algal growth, or other factors that may reduce the ecological health of a waterway (U.S. Environmental Protection Agency 2010). The goal of this list is to remediate impaired waters and remove them from this list.

Many areas of the USA contain impaired waterways. In 2016, the US Environmental Protection Agency (EPA) listed 42,509 impaired waterways on the 303(d) list due to aforementioned impairment. Cumulatively since 1995, 69,486 TMDLs have been assigned to water bodies, of which 13,313 are for high pathogen (e.g., fecal coliform) loads, 6235 for excessive nutrient loads, 3950 for excessive sediment loads, and 1351 for pesticides (U.S. Environmental Protection Agency 2016). Although agriculture is not the sole contributor to impairment in these impaired waterways, reducing the environmental impact of agriculture via non-point source contaminant reduction should be a conservation goal.

## Water Runoff and Capture

Runoff from specialty crop container operations is from two sources: uncontaminated water and operational water. In this context, uncontaminated water is water from rainfall events that has not come into contact with production areas, crops, agrichemicals, retention basins, or runoff collection reservoirs that collect and retain production runoff, nor should it contain contaminants (nutrients, pesticides, pathogens, etc.) above background levels (the level of the contaminant in nearby surface water or groundwater). Runoff from a greenhouse roof is an example, as this water should not require treatment prior to leaving an operation or mixing with operational water to supplement the irrigation water supply. Operational water is any water (i.e., rain and irrigation) flowing from, in, through, or around production areas. As a result of contact with soils, agrichemicals, and phytopathogens, this water may have elevated concentrations of contaminants, which may require treatment before reuse or release, depending on operational needs and local regulations.

### Feasibility and Limitations

Ideally, both operational water and uncontaminated water would be captured, treated, and released from or reused by container operations. This is not always possible for nursery or greenhouse operations for a number of reasons. Often, operations have geographic limitations that constrain their capacity to capture runoff. Rainfall events in some regions of the USA are intense over short durations, resulting in runoff volumes that exceed the capacity of existing containment infrastructure. In some parts of the country, a high water table can limit feasibility to capture or treat runoff water. Saltwater intrusion and storm surges are also major concerns, particularly in coastal areas (Park and Aral 2004). Some operations, especially smaller or more urban operations, may be land limited, so there may not be sufficient land area to store water for treatment or reuse. Other areas may not be able to store water due to topography or soils (i.e., rock, sand). These limitations must be considered when developing regulations and implementing BMPs for a particular area or operation.

Regulations may also limit the ability of specialty crop operations to store water. As populations increase, particularly in the western USA where water is more limited, state and local regulations may limit the amount of water that can be captured or stored at an operation. For example, Oregon requires all users, including nursery and greenhouse operations, to obtain water rights permits to store rainfall in a containment reservoir since it is considered a state resource (Oregon Water Resources Department 2013). Similar regulations may become more common across the country as water becomes more limited and may be a short-term advantage to producers not under those restrictions.

### Water Infrastructure

**Fig. 1:**
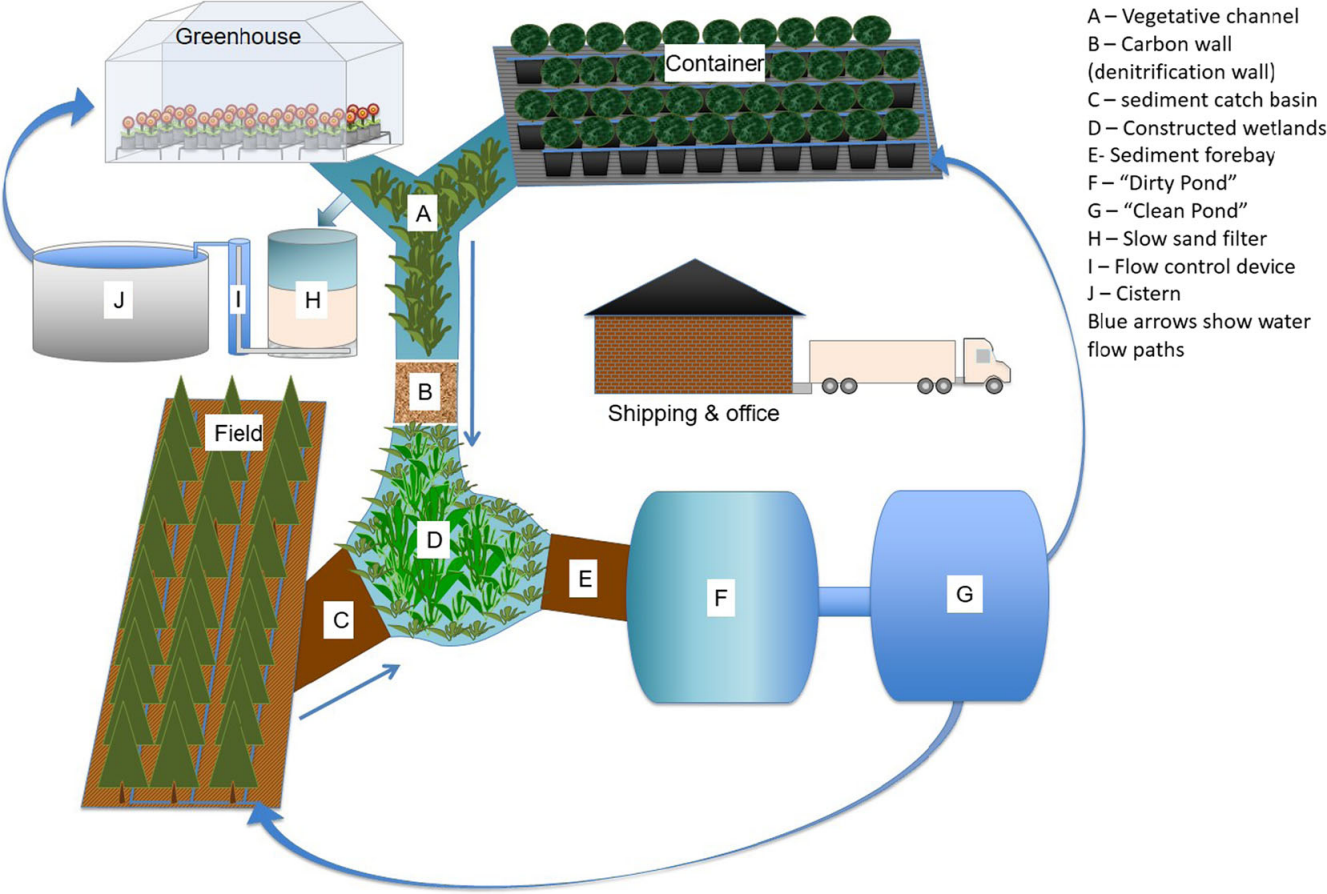
Hypothetical ornamental production operation showing ideal location of containment reservoirs, treatment trains, and other recommended management practices. All rainfall and irrigation runoff should flow to containment reservoir. Note that container and greenhouse production flow through vegetated buffer and field production areas, which can in part be used to treat nutrient runoff and other contaminants

The following information about layout and site design is meant to represent the ideal production scenario; however, site constraints and owner priorities will dictate what is possible. A new operation should be designed to balance water collection, water storage, and production to ensure ample amounts of quality water. Containment reservoirs should be situated at the lowest part of the nursery, allowing water to flow freely towards the containment reservoir while minimizing contact with production areas (Fig. 1). Chen (2011) reported remediation benefits associated with a multi-reservoir design, where water flows through multiple reservoirs before it is recycled. Pathogens are relatively short-lived without a host; therefore, if multiple ponds are used to increase water retention time, fewer pathogens survive to reinfest plants (Chen 2011). If multiple reservoirs are not available, locating the irrigation pump intake as far from the entrance of operational water as possible in order to increase hydraulic retention time and 1 m (3 ft.) above the bottom of the reservoir can help reduce pathogen loads applied to crops (Hong et al. 2009). In greenhouse operations, one or more cisterns may be used to store irrigation runoff (return water), particularly for ebb and flood systems. Return water must be treated prior to storage or reuse to reduce or remove pathogens, particulates, and other potentially harmful constituents that can impact the irrigation system and plants.

One of the most important steps to ensuring efficient capture of runoff water is proper grading and utilization of well-drained bed base such as coarse gravel. These measures can (1) reduce disease incidence by minimizing standing water under containers (Braman et al. 2015; Raudales et al. 2014) and (2) convey water to containment reservoirs for reuse or remediation (Ross 2008b). Grading may be minor or extensive, depending on the layout of the property and the site design. More detailed information regarding infrastructure and surface water recycling is available in Bilderback et al. (2013); Merhaut (2008); Yeager (2008).

## Remediation Technologies

Remediation can be defined as the process of removing chemicals, pathogens, and other constituents of concern to reduce loads of harmful substances to a water system (Kabashima et al. 2004). Contaminant type, required load reduction, and the economics and efficacy of treatment technologies depend on a number of factors at each operation. Below, we highlight research that evaluates various treatment technologies and assess where technologies may be of most effective use in production systems. A summary of each technology, scalability, relative cost (initial and continuing), contaminants managed, and relative efficacy for each technology are presented in Table 1.

**Table 1:** Scalability, cost, contaminants removed, and remediation efficacy of various treatment technologies evaluated in peer-reviewed literature

Treatment technology	Scalability^a^	Cost^b^	Particle/filter pore size	Contaminants managed	Removal efficacy (%)	References
Rapid sand filtration	Small–large	$	125 to 640 μm	Particulates	59.6–85.4%	Elbana et al. 2012; Raudales et al. 2014; Roberts and Hern 1993
Activated carbon filters	Small–large	$$ to $$	10 to 100 μm	Organic compounds and negatively charged ions	98–99.5%	Pan and van Staden 1998; Thomas 2008
Polyacrylamide	Small–large	$	NA	Sediment	80–99%	Bjorneberg and Lentz 2005; Entry and Sojka 2003; Kabashima et al. 2004; Oliver and Kookana 2006a,b; Sojka et al. 2007
				Pesticides	38–84%	
				Nitrogen	56–86%	
				Total phosphorus	79–98%	
Compost filter socks	Small–large	$	Particle size distribution^c^	Sediment		Faucette et al. 2008
				Load	68–90%	
				Concentration	62–87%	
				Nutrients		
				Total phosphorus		
				Load	14–27%	Faucette et al. 2009
				Concentration	59–65%	
				Soluble phosphorus		
				Load	14–27%	Shipitalo et al. 2010
				Concentration	55–65%	
				Pesticides	5 to 18%	
Reverse osmosis	Small	$$$	<0.5 nm	Salts, pathogens	100%	Raudales et al. 2014
Membranes:	Small–large	$$ to $$$				Ehret et al. 2001; Raudales et al. 2014; Schuerger and Hammer 2009; Moens and Hedrickx 1992; Stewart-Wade 2011
Ultra-filtration			100–10,000 nm	Suspended particles, bacteria, pathogens,	44–100%	
Micro-filtration			2–100 nm	Metals/multivalent ions	100%	
Nano-filtration			0.5–2 nm		100%	
Chlorination	Small–large	$	NA	Pathogens	7–100%	Beardsell and Bankier 1996; Lewis Ivey and Miller 2013; Newman 2004; Raudales et al. 2014
Ozonation	Small–medium	$$ $0.01–0.05 per 1000 L	NA	Pathogens (*Alternaria*, *Fusarium*, *Phytophthora*, *Pythium*) and organic matter (algae)	90–100%	Beardsell and Bankier 1996; Raudales et al. 2014; Zheng et al. 2014
UV light	Small–medium	$$ $0.01–0.05 per 1000 L	NA	Pathogens (*Alternaria*, *Colletotrichum*, *Fusarium*, *Phytophthora*, *Pythium*, bacteria, and nematodes)	15–100%	Amsing and Runia 1995; Beardsell and Bankier 1996; Raudales et al. 2014; Runia 1994; Stewart-Wade 2011; Zheng et al. 2014
Ionization:	Small	$$	NA	Algae	99% (4 mg/L Cu)	Mohammed-Pour et al. 2001
Copper				Pathogens	0–100% (0.28 to 4 mg/L Cu)	Colburn and Jeffers 2010; Granke and Hausbeck 2010; Raudales et al. 2014; Slade and Pegg 1993; Van Os 2010
Silver					99–100% (25 to 100 μg/L Ag)	
Slow filtration	Small–medium	$$	Effective size:	Pathogens (*Cylindrocladium*, *Fusarium*, *Phytophthora*, *Pythium*, *Thielaviopsis*)	89–100%	Nyberg et al. 2014; Lee and Oki 2013; Wohanka et al. 1999
Sand			0.15 to 0.3 mm (100–50 mesh)			
Pumice			Uniformity coefficient:	Pesticides	41–85%	Casas and Bester 2015
Rockwool			<3.0	Nutrients	94–99%	Aslan and Cakici 2007
Crushed brick			Silt content: <1%	Mycrocystin	>80%	Bourne et al. 2006
Constructed wetland:	Medium–large	$$ to $$$	NA	Nutrients		Taylor et al. 2006; White et al. 2010
Surface flow				Nitrogen	71–95%	White 2013b
				Phosphorus	−40–53%	Vymazal and Březinová 2015
				Pesticides	24–97%	
Constructed wetland:	Medium–large	$$ to $$$	NA	Nutrients		
Subsurface flow				Nitrogen	85–99%	White 2013b
				Phosphorus	33–90%	Narváez et al. 2011
				Pathogens	99–100%	Gruyer et al. 2013
						Headley et al. 2005
				Pesticides	24–97%	George et al. 2003; Vymazal and Březinová 2015
Constructed wetland:	Small–medium	$$ to $$$	NA	Nutrients		
Floating				Nitrogen	25–89%	Lynch et al. 2015; White and Cousins 2013; Zhang et al. 2015
				Phosphorus	4.0–78.5%	
Vegetated buffers	Small–large	$	NA	Nutrients		
				Nitrogen	47–100%	Dorioz et al. 2006
				Phosphorus	−64–93%	
				Pesticides	27–99%	Krutz et al. 2005; Otto et al. 2008
				Sediment	40–100%	Dorioz et al. 2006; Lambrechts et al. 2014; Liu et al. 2008
Denitrification bioreactors:		$ to $$		Pesticides		
Artificial media	Small–medium	$2.39–15.17 kg^−1^ N		Nitrate-nitrogen	80–90%	Wilson and Albano 2013
Carbon media	Small–large	$0.79 kg^−1^ N		Nitrate-nitrogen	50–60%	Ghane et al. 2016; Long et al. 2011; Schipper et al. 2010
Algal turf scrubbers	Medium–large	$		Nutrients	250 mg/m^2^/day N 45–730 mg/m^2^/day P	Mulbry et al. 2010; Craggs et al. 1996

^a^Scalability of treatment technology based on land area treated or volumes of runoff. Small scale (bed or greenhouse scale), medium scale (several beds/growing areas), and operation scale (or scalable to entire operation)

^b^Estimated cost of treatment technologies: $ = low, $$ = moderate, $$$ = high, depending upon both initial capital costs and investment costs, when available cost/unit treated are reported

^c^Particle size distribution determined by passing substrate through a series of mesh sieves: >25 mm (0 to 12.4%), 15 to 25 mm (14.1 to 16.1%), 9.5 to 16.0 mm (28.2 to 44.8%), 6.3 to 9.5 mm (13 to 21.8%), 4 to 6.3 mm (6.3 to 9.8%), 2 to 4 mm, (4.7 to 7.2%), and <2 mm (9 to 17.8%)

### Physical Filtration

Filtration is accomplished via several mechanisms including adhesion (one material being bound to another), flocculation (chemical precipitation), impaction (fill up a container), interception (remove from a system), and straining (filter out) (Levine et al. 1985). Contaminant removal efficacy is in part determined by particle size, contaminant loading rate, and flow rate; these should be considered when selecting treatment technologies. Important considerations for filtration include both the flow rate and the loading rates of contaminants that must be removed, as well as the cost of installation and upkeep (including parts and labor).

#### Rapid Filters

Rapid sand and glass filters consist of tanks that hold sand or glass of a specific particle size (Hudson 1963). As water moves through the sand or glass, particulates are removed. These filters are able to process large volumes of water quickly (Stewart-Wade 2011). As sand or glass particle size decreases (typical particle sizes range from 125 to 640 μm), filters are able to remove smaller particles, but require more force (larger pumps) to move the same volume of water per unit time. When the pores become clogged with particulates, the pressure required to force the water through the substrate increases, therefore treated water volume is reduced unless pressure is increased. These systems must be back-flushed (water run in reverse) to remove collected particulates and maintain the effectiveness of the filter (Elbana et al. 2012). Smaller particle size filter media clog more easily and require more frequent back-flushing. As the amount of particulates in the intake water increases, so does the frequency of backwashing, which can waste water if it is not recaptured by the system. These systems do not remove most chemical and biological contaminants, but are mainly used to limit clogging of irrigation lines and emitters and to minimize inactivation of sanitation chemicals (e.g., chlorine) via sorption to non-target particulates (Stewart-Wade 2011).

#### Mechanical Filters

Disc filters are mechanical filters that typically handle smaller volumes of water per unit time than rapid sand filters (Bilderback and Lorscheider 2007). Disc filters can remove particles up to 150 μM and are used as primary filters when water is relatively free of particulates or if only small volumes need to be treated; when larger particulates may cause clogging (i.e., mist, drip, and other micro-irrigation situations), they can also serve as secondary filters behind rapid sand filters (Dickenson 1997; Ross 2008a). Like rapid sand filters, they must be backwashed periodically to clean particulates out of the discs. Disc filters do not remove chemical contaminants or most pathogens from the water.

Other types of mechanical filters are used for specific situations. Paper filters and rotary screens are typically used to remove sediment and large debris from water that is not typically under pressure. These systems are generally used in greenhouses to filter recaptured water from the operation.

#### Activated Carbon Filters

Activated carbon is not a stand-alone treatment and should be paired with another filtration system to increase treatment efficacy (Kabashima et al. 2004). Activated carbon has a large, porous internal surface area (500 to 2000 m^2^/g) with filter pore sizes ranging from 10 to 500 μm (Pan and van Staden 1998) and can be manufactured to desired particle size with a low acid/base reactivity. Activated carbon is positively charged and can adsorb organic, moderately polar compounds and negatively charged contaminants (i.e., chloramines) depending upon source material and pyrolysis, oxidation, and purification methods (Merhaut 2008). The internal structure of activated carbon influences its capacity to adsorb contaminants (Pan and van Staden 1998). Activated carbon is used extensively in micro-propagation (tissue culture) applications to mitigate effects of inhibitory compounds on plantlet growth (Pan and van Staden 1998; Thomas 2008). As the volume of water per unit time increases, carbon filters become less effective because contact time with the activated carbon decreases. Water pH, ions present (e.g., KI, KCl, and NaCl), and concentration of other contaminants also influence the efficacy of carbon filters (Chen and Wang 2000). Activated carbon systems require periodic maintenance including replacement or regeneration of carbon once it has been saturated, which depends on water volume and contaminant loads.

Carbon filters remove some pesticides (including some herbicides) (Kabashima et al. 2004; Merhaut 2008). Economic losses associated with stunted or deformed non-target crops can be attributed to presence of residual ancymidol or paclobutrazol at concentrations as low as 3 or 5 μg L^−1^, respectively (Million et al. 1999). Detection (Altland et al. 2015) and remediation (White et al. 2014) of plant growth regulators (e.g., ancymidol, paclobutrazol) with activated carbon are currently being evaluated. The cost of the technology, along with its potential to remove beneficial compounds such as residual metals applied as fertilizer, may make its application less useful in some circumstances. Additional research on efficacy and economics of carbon filters would benefit growers, particularly in the area of plant growth regulator removal, which is a concern particularly in greenhouses.

#### Pressure-Driven Membrane Filters

Membrane filters work by exerting pressure on water on one side of a membrane to sieve particles from the water stream (solvent). The permeate, or filtered water, is pushed through the filter while the retentate, or concentrated waste stream, must be disposed of or treated (Van der Bruggen et al. 2003). Membrane filters facilitate removal of contaminants with particle sizes ranging from 0.1 to <0.0005 μm (Van der Bruggen et al. 2003; Zhou and Smith 2002). Within this size range, filter classifications are defined by pore size and membrane pressures as identified in Table 2.

**Table 2:** Membrane filter characteristics and particles excluded for treatment of irrigation water in greenhouse production

Classification	Pore size (nm)	Membrane pressure (MPa)	Particles excluded
Micro-filtration	100–10,000	0.03–0.3	Suspended solids
Ultra-filtration	2–100	0.05–0.5	Macro-molecules, bacteria, and viruses
Nano-filtration	0.5–2	0.5–1.5	Multivalent ions and organic micro-pollutants
Reverse osmosis	<0.5	5–8	Monovalent ions

Particles excluded include all materials found in rows above a specified row (i.e., nano-filtration particles excluded include all particles listed in both ultra- and micro-filtration). Additional information on membrane filtration can be found in Stewart-Wade 2011; Van der Bruggen et al. 2003; and Zhou and Smith 2002.

The pore sizes of material from which membranes are derived (ceramic, mineral, organo-mineral, or polymeric) differ and thus influence their applications and the types of contaminants that can be controlled. Membranes can become clogged over time and may require periodic remediation (weekly to yearly) to manage fouling. Remediation may consist of backwashing for micro-filtration (MF) and ultra-filtration (UF) or use of acid or alkaline detergents to mitigate inorganic or organic fouling, respectively (Van der Bruggen et al. 2003). Membranes can be produced which avoid fouling by using pre-filters or membrane surface modifications, whether to alter hydrophobic/hydrophilic ratios for nano-filtration (NF) and reverse osmosis (RO) or to manage electrostatic attraction sources so membranes actively repel fouling agents (NF, UF) (Van der Bruggen et al. 2003).

Contaminant remediation using membrane filters is considered prohibitively expensive for use in most container production systems except in asexual plant propagation of high value crops—where high-quality water is critical, due to installation and maintenance costs, pumping costs, downstream processing costs, and rapid clogging of filters (Stewart-Wade 2011).

#### Polyacrylamide

Anionic, water-soluble polyacrylamide (PAM) are long chains of linked acrylamide (C_3_H_5_NO). They have been used since 1995 as an additive to reduce irrigation-induced sediment loss, promote infiltration, and induce flocculation and aggregation of suspended solids from irrigated production runoff (Kabashima et al. 2004; Sojka et al. 2007). The PAMs used in agriculture contain less than 0.05% of acrylamide monomer, which is considered toxic to humans (Sadeghi et al. 2016; Sojka et al. 2007). Anionic PAMs are considered safe in the environment, as they have a low aquatic toxicity in comparison with cationic and non-ionic forms (Sojka et al. 2007).

Use of PAMs has primarily focused on mitigation of erosion, but when PAMs are used to flocculate suspended solids from water, they also remove any bound pesticide, phosphorus, and microbial residues that are adsorbed to those particles. Pesticide removal depends upon the chemistry of the compound; efficiencies depend upon the compound evaluated with removal averaging 78.7% for bifenthrin, 38% for bupirimate, 49% for atrazine, 49% for chlorothalonil, 54% for endosulfan, 84.2% of cis-permethrin, and 71.2% of trans-permethrin (Kabashima et al. 2004; Oliver and Kookana 2006a), though it is difficult to differentiate between flocculation of sediment-bound pesticides and pesticide removal by PAM alone. Dissolved reactive phosphorus was not removed, but particulate P was (Oliver and Kookana 2006b). Sojka and Entry (2000) reported that PAM treatment reduced total algal, bacterial, fungal, and microbial biomass in irrigation water.

Applying PAM during irrigation with rates as low as 1–2 kg/ha halted 94% of erosion from irrigated furrows (Lentz and Sojka 1994) and 92.9% sediment reduction when PAM was injected (dripped) at 10 mg/L into nursery production runoff (Kabashima et al. 2004). Estimated cost per acre in 2008 was $10 to $30 per acre at these application rates (Taliaferro and Stewart 2008).

#### Filter Socks

Filter socks are used primarily as a sediment trap or to retain some chemicals (e.g., phosphorus, oil) from construction site runoff, but in recent years filter socks have also been evaluated for mitigating sediments and agrichemicals from surface water runoff in agricultural fields (Shipitalo et al. 2010). Filter socks can be filled with a variety of organic media, primarily composted wood chips, that can be further amended with inorganic adsorbents/precipitants or synthetic additives such as PAM or other polymers to enhance flocculation, depending on their purpose (Faucette et al. 2008). Filter socks cost between $3.50 and $15.00 per linear foot for continuous non-amended or amended filter socks, with amended socks having higher costs. Filter socks can provide significant sediment control when installed and maintained properly (Faucette et al. 2009). Most filter socks have a relatively short lifespan of a few months to a year before they begin to saturate, break down, and lose their effectiveness; however, there are reusable/refillable “flexible filter hand bags” for catch basins which have been recently introduced (Patent, US 9162169 B1). Filter sock flow-through rate, and subsequent ponding prior to the sock, is affected by substrate packing density and particle size (Keener et al. 2007). If sediment levels build up to the point where water crests the filter sock instead of flowing through it, the filters are less effective. Routine inspection and cleaning of the area in front of a filter sock is important. Also, if the socks have poor contact with the ground, the volume of water is too large, or the slope is too steep, the filter sock can be bypassed, reducing treatment effectiveness.

Hydraulic flow-through rate may better predict sediment and phosphorus removal than particle size distribution alone (Faucette et al. 2008). However, substrate particle size influences hydraulic flow-through rate; thus, both flow rate capacity and particle distribution are pertinent factors when designing filter socks for sediment control. Average removal percent efficiency of compost filter socks varies by contaminant and initial concentration or load (Table 1), with concentration reductions reported for sediment (59 to 65%), total and/or soluble phosphorus (59 to 65%), or pesticides (5% for dissolved glyphosate, 21% for aminomethylphosphonic acid (AMPA), and 18% for alachlor) (Faucette et al. 2008; Shipitalo et al. 2010). Flow rate capacity and lifespan of filter socks are especially pertinent in nursery production areas, where uncontaminated water and production runoff events often flood roadways. The capacity of the filter socks to manage sediment, while remaining in place and not backing up water into production areas, is a critical design factor and requires further investigation. One option to investigate is alternate layouts that capture sediment without impeding flow, similar to stream restorations (Oregon Department of Forestry 2010).

### Chemical and Ultraviolet Disinfection

The first step in irrigation water treatment is typically physical removal of macro-particles via filtration, followed by the addition of chemical disinfectants to reduce the spread of water-borne diseases. Chemical applications are more effective when carbon-based particulates are removed prior to chemical treatment, because organic compounds create a demand on active ingredient of chemical treatments (Raudales et al. 2014). Chemical treatment efficacy declines if high levels of organic matter are present in water, necessitating higher concentrations of chemical to treat the same volume of water (Fisher et al. 2013). Whereas filtration removes physical impurities from irrigation water that are larger than single-celled microbes, chemical treatment is targeted towards the removal of biological contaminants (pathogens). Removal of pathogens and other biological organisms (algae, biofilms, etc.) improves crop health and system longevity. This section covers some of the more commonly adopted disinfection technologies including chlorine, copper, peroxides, silver, ozone, and ultraviolet (UV) light. A review of a wide range of treatment technologies not discussed herein (e.g., chlorine dioxide, quaternary ammonium chlorides, and heat) can be found in Raudales et al. (2014).

#### Chlorination

The most commonly used chemical treatment is chlorination either as a solid (calcium hypochlorite), liquid (sodium hypochlorite or hypochlorous acid), or gas (chlorine) or generated through an electrolysis process. The presence of 0.5 to 2 ppm of free chlorine at the sprinkler head is recommended to ensure adequate sanitation (Raudales et al. 2014). Chlorine levels should be routinely checked during crop production, as changes in water quality and the amount of organic matter in treated water impact the chlorine residual that will exit the sprinkler head. Hypochlorous acid (HOCl), the most important sanitizing form of dissolved chlorine, is favored over the weaker hypochlorite form at pH below 7.5. Therefore, acidification of irrigation water is often desirable prior to chlorine injection to increase chlorine efficacy. Many operations use chlorine for disinfection because of its cost effectiveness and relative ease of use. The major concern with chlorine is the additional safety precautions that are required for its use, which vary by type of chlorine. Extensive discussions related to efficacy, dose, costs and benefits, and timing of chlorine injection are available in Newman (2004), Raudales et al. (2014), and Stewart-Wade (2011).

#### Ozonation

Ozone (O_3_) is a strong oxidant that disinfects by producing a reduction-oxidation reaction in pathogens and other organic constituents (Stewart-Wade 2011). An ozone production system (corona discharge or plasma discharge units) uses electricity to split oxygen (O_2_) molecules to form ozone (Newman 2004). Ozone breaks down into peroxides and other oxygen radicals, providing additional disinfection. No additional inputs are required, and no persistent by-products are produced. Ozone and by-products degrade quickly in water, so direct testing is difficult, but in-line monitors that also control injection concentration are typically employed (Water Education Alliance for Horticulture 2015; Zheng et al. 2014). Ozone activity is reduced in the presence of organic matter, high pH, and/or high concentrations of nitrite, manganese, iron, or bicarbonate (Stewart-Wade 2011; Zhou and Smith 2002).

For a high level of disinfestation, Runia (1995) reported that a dose of 10 g O_3_/m^3^ water with a 1-h contact time at a pH 4 resulted in kill of 99.9% of bacteria and fungi. However, this process requires injecting ozone into a storage cistern rather than in-line injection, as can be used for other chemical treatment technologies. Other studies with ozone have varied contact times (2 to 30 min), with effective control achieved with ozone doses ranging from 0.01 ppm O_3_ for algae control to 1.6 ppm O_3_ to control *Phytophthora cinnamomi* chlamydospores and 1.75 ppm O_3_ to kill *Fusarium oxysporum* conidia (Stewart-Wade 2011). Ozone is one of the most expensive water treatment options in terms of installation cost, with electricity being the main operating cost (Zheng et al. 2014). Potential human health effects from ozone exposure require fail-safes and adequate venting, thus reducing the popularity of ozone for treating pathogens in irrigation water (van Os et al. 2012).

#### Peroxides

Peroxides [hydrogen peroxide (H_2_O_2_) and peracetic/peroxyacetic acid] also produce reactive oxygen (O_2_) molecules when added to water and can be added directly to irrigation lines. Peroxides can also be used in conjunction with other treatments, such as ozone and UV, to increase their effectiveness (Raudales et al. 2014). Peroxides are not generated on-site, and the chemical must therefore be purchased on an ongoing basis. Peroxides and other oxidants are corrosive. Therefore, irrigation pipes and structures where peroxide-treated water is applied should be corrosion-resistant to avoid costly replacement of irrigation components (Zheng et al. 2014). The concentration of hydrogen peroxide in an irrigation system can be monitored using inexpensive test strips (Nederhoff 2000).

#### Ultraviolet Light

Ultraviolet light is an in-line treatment that uses UV-C radiation wavelengths (240 to 280 nm) to kill pathogens and micro-organisms. High- or low-pressure UV lamps emit radiation, disinfesting recycled water. Radiation from high-pressure lamps is less energy efficient (10% power consumption converted to UV-C radiation) compared with radiation using low-pressure lamps (≈40% of power consumption converted to UV-C radiation) (Newman 2004; Runia 1995). At 254 nm, the DNA and RNA of a micro-organism is photochemically altered after absorption, destroying the organism (Stewart-Wade 2011; Zheng et al. 2014). Disinfestation efficacy depends upon exposure duration and intensity. The requisite UV-C radiation dose depends upon the target organism. Bacteria are destroyed with a UV-C dose of 3.5 to 26.5 mJ/cm^2^ (Raudales et al. 2014). Viral contaminants (tomato mosaic virus) are destroyed with a UV-C dose of 100 to 277 mJ/cm^2^ (Stewart-Wade 2011; Zheng et al. 2014). The propagules (spores, zoospores, conidia) of fungal organisms (*Alternaria*, *Colletotrichum*, *Fusarium*) and oomycetes (*Phytophthora* and *Pythium*) that can infect plants are destroyed with UV-C doses from 10 to 70 mJ/cm^2^ (Runia 1994; Stewart-Wade 2011; Zheng et al. 2014). Root infection by nematodes was prevented at 100 mJ/cm^2^, with organism death at 500 mJ/cm^2^ (Amsing and Runia 1995; Stewart-Wade 2011).

Water clarity is the single most important factor regulating UV light water disinfestation efficacy. Organic matter and other particles (turbidity) in the water absorb, reflect, and/or attenuate UV light. If using UV light to disinfest recycled water, it should be paired with a physical pre-filter treatment (e.g., sand, glass, disc, etc.) to remove turbidity. Turbidity, as defined by nephelometric turbidity units (NTUs), should be less than 2 NTUs to facilitate adequate treatment of micro-organisms (Zheng et al. 2014). Under low flow (18 L/h) and low turbidity conditions, these systems effectively disinfest plant pathogens from water (Masschelein 2002). However, the volume of water requiring treatment can range from 30,000 L/day for small greenhouse operators to more than 4,800,000 L/day for large nurseries (Chen et al. 2003). As a result, scalability and high initial investment costs may limit adoption, even though costs per 1000 L water used are relatively low (Zheng et al. 2014).

#### Copper and Silver Ionization

Hydrolysis of copper or silver via an ionization system delivers copper ions to treat biological contaminants in water (Raudales et al. 2014). Copper or silver ions can bind to protein prosthetic groups, disrupting protein structures to destroy pathogens (Raudales et al. 2014). Release of copper or silver from ionization systems can assist in biofilm growth control in irrigation infrastructure (i.e., pipes, lines) (Raudales 2014). Once installed, ionization systems are often low maintenance and cost effective. Greenhouse operations commonly use ionization systems to manage biological contaminants in water. Copper and silver could be considered pollutants of local waterways if present in runoff leaving the operation in sufficient volumes. The EPA safe drinking water standard is 1.3 mg L^−1^ for copper and 0.1 mg L^−1^ for silver. Copper and silver concentrations deemed effective for biological disinfestation range from 0.28 to 4.0 mg L^-1^ and from 0.07 to 0.5 mg L^-1^, respectively. It should be noted that phytotoxicity can occur at concentrations of copper ranging from 0.19 mg L^-1^ (*Capsicum annum*, pepper) to <1.05 mg L^-1^ (*Cucumis sativus*, cucumber). To minimize potential for non-target crop damage, phytotoxicity tests were performed on a small group of plants prior to use of water sanitized via copper or silver ionization (Zheng et al. 2014).

### Biological and Ecological Treatment

Biological- or ecotechnology-based treatment systems use “natural systems to solve environmental problems” that cannot readily be solved by use of mechanical or chemical technologies alone (Mitsch et al. 2001). Because biological components contribute to remediation efficacy, treatment efficacy may vary as environmental and physical factors influence health and growth of microbial and plant communities. Thus, some uncertainty is intrinsic to the use of these systems with regard to treatment efficacy.

#### Slow Filters

Slow sand filters have been used since 1804 to cleanse contaminants from water for both drinking water and industrial uses (Ellis and Wood 1985) and have been adopted by the European horticultural community to remove phytopathogens from reused irrigation water since the early 1990s (Stewart-Wade 2011; Ufer et al. 2008; Wohanka et al. 1999). Slow filters consist of three major components: the underdrainage, gravel, and a sand (or substrate) layer. The underdrainage supports the gravel and sand layers while facilitating complete drainage of water through the system. The gravel supports the sand bed while preventing sand from clogging the underdrainage. The sand bed (0.6 to 1.4 m deep) facilitates the purification process. Water flows through these sections via gravity. The water reservoir above the sand layers should be 1.0 to 1.5 m in depth to maintain desired head pressure that ensures consistent water flow through the sand filter.

Recommended filtration rates range from 2.0 to 5.0 m^3^/day m^−2^ of surface area (Huisman and Wood 1974). Faster flow rates may achieve desired quality standards, but the filters are likely to clog both more deeply and quickly, requiring more frequent maintenance to assure filter functionality. The capacity of a filter to achieve treatment standards at faster flow rates depends upon the quality of the water entering the filter (feed water). If the feed water has been pre-filtered to remove turbidity or other organic materials, higher flow rates are feasible with filtrate meeting desired quality standards.

A biologically active filter skin layer begins to form at the water/media interface (top few centimeters), when water flows through the filter (Ellis and Wood 1985). The filter skin is populated by micro-organisms (e.g., actinomycetes, algae, bacteria, bacteriophages, diatoms, fungi, plankton, protozoa, rotifers, etc.) that assist with the straining process, helping to trap and degrade agrichemical and pathogen contaminants via aerobic biological processes (Calvo-Bado et al. 2003; Hunter et al. 2013; Oki and White 2012).

Contaminants are removed both by physical straining and micro-organism breakdown within the biologically active layer. Remediation efficacy depends on the particle size of the substrate, the diameter of the contaminant to be removed, and the biological activity within the skin layer. For contaminants >2 μm in diameter, physical processes likely drive removal (Wohanka et al. 1999), while for contaminants <2 μm in size, biological processes enhance removal efficacy (Erwin and Ribeiro 1996; Nyberg et al. 2014). Various fungal (*F. oxysporum* f. sp. *cyclaminis*, *Cylindrocladium* sp., *Phytophthora capsici*, *P. cinnamomi*, *Phytopthora nicotinanae*, *Pythium* sp., and *Thielaviopsis* sp.), bacterial (*Xanthomonas campestris* pv. *pelargonii*), nematode (*Radopholus similis*), and viral (tobacco mosaic virus) contaminants were removed via flow through a slow filter, using beds composed of various substrates such as sand, rockwool, pumice, or crushed brick (Lee and Oki 2013; Nyberg et al. 2014; Stewart-Wade 2011; Wohanka and Helle 1997; Wohanka et al. 1999).

A pilot system should be established to determine capacity flow rate and required substrate bed and water depth (Ellis and Wood 1985). Periodic maintenance (i.e., raking and/or removal of impacted depth of substrate) is required to maintain design flow rates through the slow filter. Installation of two slow filters in parallel is recommended for horticultural applications (Oki and White 2012). Paired installation allows for continuous treatment capacity as one filter is taken off-line for maintenance. One month prior to filter maintenance, water recycling was started through the alternate filter so microbial communities can mature within the filter skin, achieving design-specified treatment capacity (Nyberg et al. 2014; Oki and White 2012).

#### Constructed Wetlands

Constructed wetlands are engineered systems that function similarly to natural wetlands in that they harness the potential of soils, aquatic vegetation, and microbial communities to remove or break down contaminants in water (Vymazal 2007; White 2013b). Three types of wetlands are used to manage runoff from plant production: surface flow (free water surface), subsurface flow, and floating treatment wetlands (White 2013b). Since sediment, plants, and micro-organisms interact to treat water, treatment effectiveness is in part dependent on environmental conditions such as temperature and rainfall, factors which are beyond grower control (Taylor et al. 2006; White 2013b). Hydraulic retention time (HRT), the amount of time it takes a unit of water to flow through the system, as well as proper and routine maintenance influence the system treatment efficacy (Vymazal and Březinová 2015). Taylor et al. (2006) and Huett et al. (2005) reported that 3.5 days HRT was adequate to mitigate N from plant nursery irrigation runoff. Typical mineral nutrient concentrations in nursery runoff range from 1.35 to 135 mg L^−1^ NO_3_-N and 0.01 to 20 mg L^−1^ PO_4_-P (Huett et al. 2005; White 2013b). Pesticide removal and/or retention within constructed wetlands is achieved by adsorption, hydrolysis, microbial degradation, photolysis, plant uptake, and sedimentation (Vymazal and Březinová 2015). Vymazal and Březinová (2015) summarized 47 studies with constructed wetlands and pesticide removal efficacy and determined that pesticide removal depended upon pesticide class [organochlorines (97%), strobilurins (96%), organophosphates (94%), pyrethroids (87%), aryloxylakanoic acids (35%), urea-based (50%), and triazinones (24%)]. Pesticide removal was mostly governed by low water solubility (high K_oc_ and K_ow_ coefficients), but in some cases solubility did not influence remediation efficacy (Vymazal and Březinová 2015).

In surface flow wetlands, water pools above the soil/sediment and flows around plant stems, where nutrients can be taken up by plant roots. Nitrogen removal in surface flow wetlands ranges from 40 to 95% and is dependent on a number of factors including the plant species used, HRT, and the concentrations of the nutrients (Taylor et al. 2006; Vymazal 2007). Phosphorus removal in surface flow wetlands is highly variable, ranging from −40% (more P exported from the system than was loaded, likely due to desorption from sediment and plant senescence) to accumulation of +40 to 50% of P loaded (Vymazal 2007; White et al. 2010). A major disadvantage of surface flow wetland systems is the land area required for adequate treatment compared with land area dedicated to plant production. Surface flow wetland sizing depends on a number of factors including the volume of water to be treated, the type and concentration of contaminants, soil type, and the types of plants that are used in the system.

In subsurface flow systems, water flows directly through a porous medium (sand, gravel, etc.), with design specifications that limit the potential for water to percolate above the substrate surface. Plants are grown in the porous medium (substrate), and water flows through the substrate and roots that are colonized by microbial communities. The major benefit of the subsurface flow wetland system is the potential to decrease treatment system surface area and yet maintain or increase treatment volume by increasing depth. In surface flow systems, plant selection and subsequent colonization is predicated by pool depth, with some species tolerating 30 cm or less and others tolerating up to 160 cm of water (Kadlec and Wallace 2008; White et al. 2012). In subsurface flow systems, plant crowns remain above the water line, and nutrient uptake into the plant relies solely on root interception. Adequate carbon must be supplied for subsurface flow systems to function efficiently, with carbon contributions from plant root systems generally being adequate to facilitate N remediation. If subsurface flow wetlands remain unplanted, an additional carbon source (e.g., molasses, methanol) is required to facilitate N remediation (Huett et al. 2005). Nitrogen removal facilitated by subsurface flow wetlands treating nursery effluent ranged in efficacy from 74 to 84%, while P removal in subsurface flow wetlands ranged from 64 to 88% (Huett et al. 2005). Pathogen (*Pythium ultimum*, *F. oxysporum*, *P. cinnamomi*) remediation in subsurface flow constructed wetlands ranged from 99 to 100% when planted with common reed (*Phragmites australis*) or cattail (*Typha latifolia*) (Gruyer et al. 2013; Headley et al. 2005).

Floating treatment wetlands (FTWs) use a buoyant scaffolding to suspend plant roots within the water column of a reservoir. Plant roots grow into the water column to remove nutrients from the water, which also allows for the colonization of micro-organisms. The major benefit of these systems is that they can be deployed in a reservoir that is currently being used to store water and therefore do not take up additional space in an operation. Also, plants grow with minimal maintenance and can adapt to the local conditions. It is important to grow floating wetlands only in reservoirs that are deep enough that plants will not root into the bottom of the reservoir during periods of low reservoir volume, typically with a recommended minimum depth of 1 m. Plants that become rooted in the reservoir will be difficult to remove and could be killed when water levels rise above the stems when the reservoir recharges. There are safety concerns with installation and removal of plants since banks are often sloped. Not all plants can be grown hydroponically, so research is needed to determine which plants will maximize nutrient uptake while requiring minimal maintenance. Another option would be for growers to utilize FTWs as production space, although there are a number of issues that would need to be overcome such as worker safety, ease of planting and harvest, determining necessary production cycle changes, and potential agrichemical interactions.

Removal efficacy for nitrogen in FTWs ranges from 25 to 89%, while phosphorus removal efficacy ranges from 4 to 79% (Lynch et al. 2015; White and Cousins 2013; Zhang et al. 2015). Plant uptake of nutrients is a pertinent mechanism driving nutrient remediation within FTWs. Plant nutrient uptake capacity varies among plants (Polomski et al. 2007); thus, plant selection for use within FTWs should be based on average influent nutrient concentration and frequency of nutrient load. Wang et al. (2015) reported that *Pontederia cordata* (pickerelweed) absorbed 7.6 mg P/plant, while *Schoenoplectus tabernaemontani* (softstem bulrush) absorbed only 1.6 mg P/plant after 5 months in a FTW with reservoir median nutrient concentrations of 0.15 mg/L total P and 1.2 mg/L total N. White and Cousins (2013) reported that *Juncus effusus* (soft rush) absorbed 31 mg P/plant and 530 mg N/plant, while *Canna flaccida* (golden canna) absorbed 20 mg P/plant and 312 mg N/plant, when median nutrient exposure concentrations were 0.16 mg/L total P and 1.2 mg/L total N over 7 months.

Plant harvest as a means to limit the potential of internal nutrient cycling is debated in published research. The debate centers on timing, frequency, cost (labor and disposal), plant species, and utility of plant harvest. Nutrient loading rate and climate (tropical or subtropical vs. temperate or arctic) contribute to the potential for plants to accumulate nutrients. Floating treatment wetlands are likely the only type of constructed wetland in which harvest will prove a sustainable practice in terms of feasibility, labor costs, and added value of plants (Tanner and Headley 2011; Wang et al. 2015; White 2013b; White and Cousins 2013). In FTWs, plants are suspended above the water column, not rooted into the substrate, thus both shoots and roots can be harvested, lending potential for an alternate use of plants either for restoration plantings, for sale to consumers, or for composting and incorporation as a soil amendment. Any of these solutions remove nutrients from the pond, limiting internal cycling of nutrients attributable to plant senescence.

Each type of constructed wetland has advantages and disadvantages associated with its use. These include cost, scalability, ease of maintenance, treatment capacity, and volume of water treated. Cost is the primary factor limiting application, because designing and installing a constructed wetland is expensive. Yet, after installation, the operational costs for surface and subsurface flow wetlands were limited to pumping expenses and periodic (typically, yearly) maintenance, making them relatively inexpensive to operate after the initial capital investment. Floating treatment wetlands are less expensive to install as they can be established in existing containment reservoirs. They may have higher maintenance costs associated with plant installation and harvest, depending on local conditions and plants selected. However, some costs associated with floating treatment wetlands could be turned into additional revenue, if the plants selected for remediation use in the floating treatment wetlands could be harvested and sold. Market demand for native, aquatic plants used for restoration and vegetative stormwater projects is increasing (Brzuszek and Harkess 2009; Clewell and Aronson 2013; Helfand et al. 2006), and floating wetland systems could be a viable solution to remediate specialty crop production runoff while producing marketable crops. Few nurseries currently market wetland mitigation plants along with common nursery crops; therefore, a thorough economic analysis of this approach is needed to determine its efficacy. Surface flow and subsurface flow constructed wetlands are easily scaled from small to large, and these systems can manage most contaminants as long as they are designed for adequate retention time. Medium to large growers will be more likely to use surface and subsurface flow constructed wetlands as economies of scale increase, with regard to treatment surface area and water volume treated. Floating treatment wetlands are scalable across operation sizes, but are limited by the surface area of the containment reservoirs and a grower’s ability to access and maintain the plants.

#### Vegetated Buffers/Channels

Operations typically convey surface water via pipes or vegetated channels either to a containment reservoir or off-site. Although pipes efficiently move water, typically no treatment occurs during conveyance. Vegetated channels and buffers on the other hand are able to provide various types of water treatment, depending on their design, construction, and maintenance. Vegetated buffers are sloped strips of vegetation that reduce the sheet flow of water (continuous flow spread over a planar surface) permitting sediment to drop out of the water. Vegetated channels are water conveyance structures that move water from one location to another. Both vegetated buffers and channels reduce water velocity, allowing sediment to drop out of the water column. Depending on subsurface characteristics (soils and water table proximity), both buffers and channels can also increase water infiltration, which is beneficial if nutrient remediation is important or if an operation would like to reduce the volume of runoff leaving their facility.

The effectiveness of vegetative buffers depends on a number of factors including the slope and width of the buffer, the volume of water to be treated, the plants used, and how well the buffer is maintained (Abu-Zreig et al. 2003; Chen et al. 2016; Sheridan et al. 1999; Wenger 1999). Vegetative buffers are most effective when sheet flow is maintained, since channelization of flow reduces retention time as well as the surface area available for sediment trapping and water infiltration. For maximum benefit, vegetative buffers should be sized for the treatment area based on the expected runoff volumes for a particular operation (see Liu et al. 2008 for a comprehensive analysis and an equation for buffer width based on slope). In short, Liu et al. (2008) reviewed 80 experiments, and meta-analyses of these data indicated that sediment removal was maximized with a 10-m buffer width and 9% slope, regardless of the ratio of buffer area to watershed area. A review of 11 studies by Dorioz et al. (2006) evaluated vegetated buffer strip efficacy for a range of contaminants which reported that remediation efficacy ranges from 47 to 100% for nitrogen, −64 to +93% for total phosphorus, and −83 to +89% for dissolved phosphorus. Phosphorus mitigation is of particular concern, since it is difficult to remove from soil and aquatic systems. Sediment removal ranged from 40 to 100%, depending upon vegetation type and growth stage, slope, and buffer width (Dorioz et al. 2006; Lambrechts et al. 2014; Liu et al. 2008). Otto et al. (2008) reported pesticide removal efficacies ranging from 81 to 99% for metolachlor and 74 to 99% for terbuthylazine (Dorioz et al. 2006).

#### Denitrification Bioreactors

##### Carbon Media

Three types of carbon-based denitrification bioreactors are commonly used to mitigate nitrate-rich subsurface runoff: beds, layers, and walls. Denitrification beds, also known as wood-chip bioreactors, are containers filled with a carbon-rich material; this type shows the most promise for management of irrigation runoff from specialty crop production areas. Denitrification layers consist of horizontal layers of carbon-rich material, while denitrification walls consist of carbon-based material installed vertically in the ground through which groundwater flows are intercepted (Ghane et al. 2015). These systems have been applied extensively in agricultural production regions throughout the world.

Bioreactors are installed using a carbon-based substrate such as bark, wood chips, mulch, sawdust, straw, or carbon from various other waste products (Bednarek et al. 2014). Carbon-rich substrates serve as both electron donors and sources of cellular material for microbial communities in the bioreactors (Bednarek et al. 2014; Ghane et al. 2015). More information is needed with regard to carbon source quality and longevity of denitrification support, as high-quality carbon sources can support denitrification for 9–15 years (Bednarek et al. 2014; Long et al. 2011). In these bioreactors, nitrogen-rich runoff water flows through a carbon-based substrate, and anaerobic conditions (dissolved oxygen <0.5 mg L^−1^) within the media promote reduction of nitrate-N to N_2_ gas.

Bioreactor cells are filled with an artificial media, typically a plastic substrate with uniform particle sizes and shapes. Artificial media provide a surface area that is colonized by microbial populations that facilitate contaminant remediation, but a supplemental carbon source is required to support growth and energetic needs of microbial communities. These systems have been used to manage nitrogen in a range of industrial applications (Ødegaard et al. 1994). Wilson and Albano (2013) used a Kaldnes media with a molasses-based carbon source to reduce nitrogen-rich irrigation runoff at a Florida nursery from 5.9–11.9 mg L^−1^ (influent) to 0.1–1.0 mg L^−1^ (effluent), an 86 to 97% reduction, when adequate carbon was supplied to the system. Unintended negative consequences of the use of denitrification bioreactors are also possible: (1) nitrate transformation may be incomplete, potentially releasing nitrous oxide (N_2_O), a potent greenhouse gas (Woli et al. 2010); (2) the release of carbon dioxide or methane may occur during degradation of organic matter (Ghane et al. 2015); or (3) methylation of mercury can occur if all nitrate is reduced and sulfate-reducing bacteria are active (Shih et al. 2011).

#### Algal Turf Scrubbers

Algal turf scrubbers (ATS) use sheet flow and a large surface area to grow algae for nutrient removal (mainly N and P). Nutrient-rich water is pumped over a shallow trough that is lined with plastic or cement. Every few days, dependent on temperature, algae are scraped off the surface, and the biomass is collected while remaining algae continue to grow. The algae uptake N and P from the water, and harvesting the algae removes N and P from the system. The harvested algae can then be used as a fertilizer, a biofuel feedstock, or otherwise as a nutrient source or soil/substrate amendment. Turf scrubbers can be easily sized from operational to watershed scale if enough land is available, with the ability to treat 40–80 million liters per day or more. Algal turf scrubbers have been found to produce 5 to 10 times the biomass of land-based systems, potentially decreasing the amount of land required for remediation (Adey et al. 2011).

In dairy operations, Pizarro et al. (2006) reported that ATS costs averaged $450 to $650 per cow per year, while dairy cows averaged $500 annual profit per animal. This assessment did not take into account any products that were sold from the algae produced, nor the environmental benefit of removing those nutrients from the environment. Mulbry et al. (2008) noted that even at $780 per cow per year for operating an ATS, that amounted to $11 for removing 1 kg of N, much less expensive than many other options such as wastewater treatment plant upgrades. At loading rates of 0.3 to 2.5 g total N and 0.08 g to 0.42 g total P per square meter per day, algae were able to produce a biomass of 2.5 to 25 g dry weight per square meter per day, of which 7 and 1% were N and P on average, respectively (Mulbry et al. 2008). Small-scale (1 m^2^) ATS were also shown to be effective when installed directly into waterways in the Chesapeake Bay watershed, remediating on average 250 mg total N and 45 mg total P per square meter per day at the most productive site, which equates to 380 kg N per ha and 70 kg P per ha, based on 150 days of operation per year (Mulbry et al. 2010). Craggs et al. (1996) found a higher rate of P removal at 730 mg per square meter per day in a similar system, which was based on an average of 2.1% P by dry weight. Optimization of ATS systems depend on a number of factors including flow rate, pulsed vs. constant flow, pH, and whether systems are run continuously or only running during the day (Sindelar et al. 2015). Additional research to address these issues for growers would be beneficial. The feasibility of ATS for nursery production will be largely dependent on the amount of land available, installation and maintenance costs, and the benefit that the operation realizes in regards to nutrient runoff reduction.

### Treatment Trains, Combinatorial, and Advanced Treatment Efforts

Although each of the treatment technologies discussed above can help reduce environmental impacts associated with production runoff, additional gains in treatment efficacy can be realized via pairing two or more types of treatment systems in series or other combinations which are synonymous with treatment train or chain. This coupling is often most effective when targeting different types of physical, chemical, and biological contaminants (e.g., agrichemicals and pathogens, or pathogens and sediment), since there is no single treatment system that will effectively manage all types of contaminants (Biswas et al. 2007; Drapper and Hornbuckle 2015; Gearheart 1999; Kabashima et al. 2004; Nyberg et al. 2014). Much of the research with treatment trains has been focused on stormwater runoff (Campbell et al. 2004) and wastewater treatment systems (Zeng et al. 2016), with fewer studies focused on agricultural water treatment trains for container-grown crop production. Kabashima et al. (2004) demonstrated treatment train efficacy at treating nursery production runoff via use of PAM injection paired with sediment traps and 340 m of vegetated buffers that increased sediment, bifenthrin, cis-permethrin, and trans-permethrin removal by 5.6% (to 98.5%), 12.1% (to 90.8%), 9.8% (to 94.0%), and 20.5% (to 91.7%), respectively. We do not know if there is a practical limit to the number of treatment options that can be applied in series, or if the order in which they are placed influences the relative efficacy of treatment for specific contaminants. These considerations need to be addressed so that science-based recommendations for treatment-train use at commercial growing facilities are available.

As environmental regulations become stricter or additional economic benefits are realized in the future, specialty crop producers will likely install water treatment technologies either singly or in treatment trains. The costs and efficacies of these treatment systems need further validation to encourage adoption of treatment technologies. On-site evaluations (case studies) of treatment system efficacy will confirm scalability and transferability of treatment efficacy across production systems. Hong and Moorman (2005) and Raudales et al. (2014) noted that little efficacy information is available for container production settings other than chlorination. Some technologies have been trialed at nursery and greenhouse operations [e.g., constructed wetlands (White 2013b); carbon filters (Altland et al. 2015; Schmidt and Clark 2012; van Os et al. 1994); PAM and sediment traps (Kabashima et al. 2004); bioreactors (Wilson and Albano 2013)], but in other instances (e.g., activated carbon, PAM) cited research has been conducted in alternate agricultural production systems or for industrial wastewater treatment (Oliver and Kookana 2006a; Skouteris et al. 2015; Sojka et al. 2007).

## Conclusions

The ability to grow plants in soilless substrates and plastic containers has produced a major shift from in-ground to aboveground production of specialty crops. Aboveground plant production has necessitated the application of large volumes of irrigation water compared with field production, which can lead to sediment loss and chemical runoff. Application of agrichemicals to modify plant growth and control pest and plant disease problems can impact the water quality of both surface water and groundwater, either on-site or in the surrounding environment if the proper precautions are not taken to protect waterways.

A number of BMPs exist to mitigate sediment and chemical runoff from agricultural production, including water recapture and reuse. Significant grower concerns exist regarding the chemical and biological contaminants that may be reapplied to plants if operational water is reused. In order to mitigate these concerns and explain the current state of information related to water remediation, we discuss a number of management practices for the remediation and reuse of water at an operation or release into the environment after treatment. Various forms of water filtration remove organic material resulting in increased disinfection efficacy (e.g., chlorine, UV light, or a number of other disinfectants). Compounds (e.g., activated carbon, and polyacrylamide) can be used to bind chemicals of concern. Sediment and any compounds bound to it can be removed in a number of ways including sediment traps and filter socks. Biological treatment options (e.g., slow sand filters, treatment wetlands, bioreactors, algal turf scrubbers, and vegetated channels) can remove physical, chemical, and biological contaminants. Treatment BMPs can be used in series or parallel, depending on operational requirements and the contaminant(s) to be removed from the water. Each BMP differs in installation and maintenance costs and has both benefits and drawbacks that need to be considered prior to implementation. In some areas, additional research is required to provide growers and consultants with rigorous science-based information related to the efficacy and longevity of treatment options.

There is the potential for more stringent regulations to be enacted internationally and in the USA at the federal, state, and local level to address water quality problems (Fulcher et al. 2016). A number of areas in the USA (e.g., California, Florida, the Chesapeake Bay watershed, the Great Lakes region, the Willamette Valley) and throughout the world (Australia, EU) have approved regulations restricting agricultural water use or runoff. Restrictions are likely to increase over time with increasing population numbers and changing climate patterns, which will strain surface water and groundwater resources. Future regulations, in terms of how much an agrichemical load or concentration must be reduced, and incentives available for implementing various practices will likely impact adoption rates of various contaminant-specific, scientifically vetted remediation BMPs. These regulations may target container-grown specialty crop production specifically or agriculture in general. It is important to have both cost and efficacy information available, so that producers can make informed decisions.

Proactive growers may voluntarily choose to remediate potential problems at their operation by replacing or repairing broken, leaking, or inefficient systems, which would reduce water use and subsequent runoff and use appropriate BMPs where possible, including water storage, treatment, and reuse facilities at their operation. It is recommended that growers document changes, including cost, to show good faith efforts to improve their operation’s environmental sustainability. Growers can be disincentivized to make changes at their operation, since these investments may not be counted in their favor if new regulations are passed. If for example, regulations are implemented requiring irrigation volumes to be reduced, they could allow waivers for growers who have documented past irrigation reductions due to better management practices.

A major goal of research is to provide information for individuals and society to make the best decisions for people and the environment. Science-based recommendations are an important foundation for mitigating water challenges of the present and the future. As regulations change, specialty crop producers will need to remain flexible, and research is needed to continue to provide viable solutions to the issues that are faced by growers, as well as to ameliorate potential environmental problems in surface water and ground water.
